# Sodium Alginate–Gelatin Nanoformulations for Encapsulation of *Bacillus velezensis* and Their Use for Biological Control of Pistachio Gummosis

**DOI:** 10.3390/ma15062114

**Published:** 2022-03-13

**Authors:** Mojde Moradi Pour, Roohallah Saberi Riseh, Yury A. Skorik

**Affiliations:** 1 Department of Plant Protection, Faculty of Agriculture, Vali-e-Asr University of Rafsanjan, Imam Khomeini Square, Rafsanjan 7718897111, Iran; moradi.mojde21@gmail.com (M.M.P.); r.saberi@vru.ac.ir (R.S.R.); 2 Institute of Macromolecular Compounds of the Russian Academy of Sciences, Bolshoi VO 31, 199004 St. Petersburg, Russia

**Keywords:** sodium alginate, *Macrocystis pyrifera*, gelatin, carbon nanotubes, silica nanoparticles, biological control, PGPR, pistachio

## Abstract

Biopolymer-based nanocomposites are favorable materials for the encapsulation of biofertilizers and biocontrol agents. In this research, sodium alginate, a widely used natural polymer, was extracted and purified from *Macrocystis pyrifera*. Its composition was confirmed using ^1^H NMR and FTIR analyses, and its molecular weight and mannuronic acid/guluronic acid ratio were obtained. Sodium alginate–gelatin microcapsules enriched with carbon nanotubes and SiO_2_ nanoparticles were prepared to encapsulate *Bacillus velezensis*, and the biological effects of this formulation on the control of pistachio gummosis and growth parameters were investigated. Microscopy examination showed that the microcapsules had quite globular shapes. XRD confirmed the occurrence of an electrostatic interaction when sodium alginate was blended with gelatin. The survival rate of the encapsulated bacteria was about 10^7^ CFU/mL and was maintained after one year of storage. The aim of this study was to achieve a unique formulation containing beneficial bacteria and nanoparticles for the synergistic control of *Phytophthora drechsleri*.

## 1. Introduction

Biopolymers are natural polymers produced by fauna, flora, and microorganisms. Many biopolymers, such as carbohydrates (e.g., sodium alginate, gellan gum, xanthan gum, chitosan, starch), proteins (e.g., gelatin, whey protein), and lipids (e.g., types of butter) [1], are useful in encapsulation technology, and carbohydrate polymers now play a key role [2,3]. The encapsulation of microorganisms is attracting attention as a method to improve the efficiency of beneficial microorganisms [4,5,6,7]. This encapsulation involves coating particles containing active microorganisms with layers of other materials that protect the viability and activity of the microbes.

Among the many biopolymers used for encapsulation, sodium alginate (ALG) is currently the most widely used [8,9,10,11]. However, the high cost of obtaining this naturally occurring polysaccharide polymer limits its commercial use. By contrast, another natural polymer, gelatin, has the benefits of low cost and biodegradability, and many researchers reported that encapsulation using a combination of ALG and gelatin is more efficient than using ALG alone [12]. Encapsulating different formulations of microbial agents using these agents can play an important role in the biological control of plant diseases.

The use of beneficial plant growth-promoting rhizobacteria (PGPR) is currently an active area of research for controlling disease and improving growth in important crop plants. In Iran, *Pistacia vera* L. (pistachio) is a main commercial agricultural product, but this crop suffers from a number of diseases. One key disease that adversely affects pistachio trees in Iran is gummosis, caused by infection with *Phytophthora drechsleri*. The arid climate of Iran also imposes drought stress, which can cause significant damage to pistachio crops [13]. The use of silica fertilizers can reduce the need for irrigation in arid areas [14]; however, the addition of beneficial PGPR can further improve plant growth under stress conditions while also overcoming the disease. Protecting the PGPR in nanoscale biopolymer coatings would therefore be expected to significantly improve sustainable agriculture. Biological control can be further aided by the use of substances that can have a synergistic effect with these biocontrol agents and improve their function. One of these is nanoparticles.

**Figure 1:**
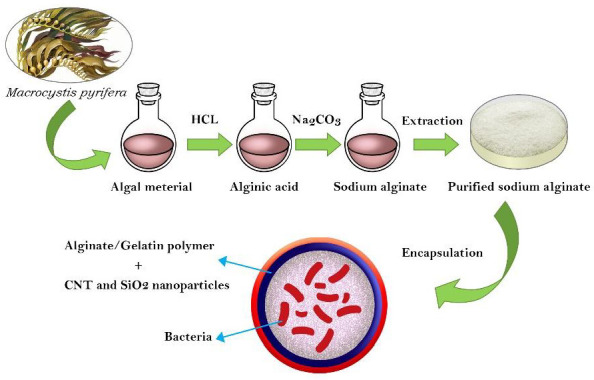
The main strategy used in the study.

Nanoscience and nanotechnology affect all areas of human life, and agricultural science is no exception [15]. Carbon nanotubes (CNTs), one of the first structures discovered in the field of nanoscience and nanotechnology, are shown to increase the rate of seed germination and plant growth [16]. The aim of the present study was to prepare an intelligent formulation based on a biopolymer/nanoparticle mixture (ALG-gelatin enriched with CNTs and SiO_2_ nanoparticles) that would provide a gradual release of beneficial bacterial (*Bacillus velezensis*) and nanoparticle agents to control pistachio gummosis, increase plant growth and resistance, and consequently improve plant yields. The controlled, gradual release of the biocontrol agent was expected to increase colonization efficiency and protect the biocontrol agent against drought stress for a prolonged period. This study is the first to use encapsulated PGPR bacteria for the biocontrol of pistachio gummosis. A scheme illustrating the main strategy of this work is shown in Figure 1.

## 2. Materials and Methods

### 2.1. Pathogen (Phytophthora drechsleri) Isolates

*Phytophthora drechsleri* isolates were obtained from the Pistachio Research Center of Rafsanjan (Rafsanjan, Iran). The identification of the isolates as *P. drechsleri* was based on their morphological characteristics.

### 2.2. PGPR Bacterial Strain

*B. velezensis* strain was obtained from the Biological Control Collections of Vali-e-Asr University of Rafsanjan (Rafsanjan, Iran). For short-term storage, the bacterial strain was cultured on nutrient agar in Eppendorf tubes. After 24 h of growth, the culture was overlayered with sterilized parafilm and stored at 4 °C.

### 2.3. In Vitro Inhibitory Potential of B. velezensis

The *B. velezensis* strain was spotted on a potato dextrose agar (PDA) medium plate, and one disc of 7- to 14-day cultured *P. drechsleri* (5 mm in diameter) was placed in the center of the plate. The plate was incubated at 27 °C, and the diameters of the inhibition zones were measured two weeks after inoculation [17].

### 2.4. Evaluation of Plant Growth-Promoting and Biocontrol Properties of B. velezensis

Indole-3-acetic acid (IAA) production by the *B. velezensis* strain was assessed by the laboratory method described by Patten and Glick [18]. Siderophore production capacity was evaluated using chrome azurol agar medium [19], and mineral phosphate solubilization activity was measured on tricalcium phosphate agar plates [20]. Protease, lipase, and cellulase production were characterized according to the methods of Ariffin et al. [21], Berg et al. [22], and Ertuğrul et al. [23], respectively.

### 2.5. Encapsulation Procedure

#### 2.5.1. Materials

Sodium alginate was extracted from brown seaweed (*Macrocystis pyrifera*) and purified in our laboratory. The soybean oil used in this research was purchased from Sigma-Aldrich. Gelatin, CaCl_2_, and Span 80 were obtained from Merck Company. CaCO_3_ nanoparticles (50 nm) and carbon nanotubes (CNTs) (purity 90%, inside diameter 10 nm, outside diameter 30 nm, length 100 nm, -COOH as a functional group) were prepared by the Iranian Nano Pishgaman Company (Mashhad, Iran) and the Faculty of Pharmacy, Kerman University of Medical Sciences (Kerman, Iran), respectively. The silica nanoparticles were synthesized in the Nanotechnology Laboratory of Vali-e-Asr University of Rafsanjan (Rafsanjan, Iran).

#### 2.5.2. Extraction of ALG

ALG was extracted from brown seaweed (*Macrocystis pyrifera*). In brief, 10 g of crushed dry seaweed was moistened with 400 mL of distilled water, and 0.1 M HCl was added under fast stirring to adjust the solution to pH 4 [24]. The seaweed was stirred in this solution at room temperature for 15 min, and then the supernatant was removed. The moistened seaweed pellets were placed in an Erlenmeyer flask containing 250 mL of 1 N Na_2_CO_3_ (pH 11.5) and stirred for 2 h at 60 °C. The extracted ALG was diluted with 800 mL of distilled water, one gram of diatomaceous earth was added, and the mixture was stirred for 15 min. The undissolved material was separated by centrifugation, leaving ALG in the supernatant phase [25].

#### 2.5.3. Purification of ALG Extracts

For purification, 250 mL of 1 M HCl was added to the extracted ALG at room temperature and stirred for 1 h. The solution was centrifuged, and the precipitate containing alginic acid was separated. The precipitate was combined with 100 mL of distilled water and 150 mL of 1 N Na_2_CO_3_ and stirred at room temperature for 1 h to produce ALG, which was precipitated by the gradual addition of ethanol (1:1, *v/v*).

#### 2.5.4. Chemical Structure of the Extracted ALG

The chemical structure of the extracted ALG was confirmed by Fourier Transform Infrared (FTIR) spectroscopy and nuclear magnetic resonance (NMR) and was compared with a commercial sample. For ^1^H NMR analysis, an ALG sample (10 mg/mL) was dissolved in D_2_O and analyzed on a Varian UNITY Inova 500 MHz spectrometer (Varian, Palo Alto, CA, USA).

#### 2.5.5. Physicochemical Properties of ALG

As a first concentration, ALG solutions (extracted and commercial, 30 mg/10 mL) were prepared by stirring for 2 h at 28 °C (room temperature). The molecular weight and density of the two samples were evaluated according to the method described by Sellimi et al. [26]. The weight average molecular weight (Mw) of the ALG was determined by size exclusion chromatography (Shimadzu Scientific Instrument Inc., Kyoto, Japan) according to the method of Ma et al. [27], and the mannuronic acid/guluronic acid (M/G) ratio was obtained by the method described by Gómez-Ordóñez and Rupérez [28].

#### 2.5.6. Synthesis of SiO_2_ Nanoparticles

The SiO_2_ nanoparticles were synthesized using the method of Li et al. [29], with some modifications. In brief, 5 g of tetraethyl-orthosilicate were added to 100 mL of distilled water and stirred until a clear solution had formed. The nanoparticle size was stabilized by adding polyvinylpyrrolidone surfactant (at 5%, based on the weight of the raw material), followed by 10 mL of 1 M NaOH. The solution was then placed in an autoclave and heated at 180 °C for 24 h.

#### 2.5.7. Evaluation of the Antibacterial Activity of Nanoparticles (SiO_2_ and CNTs)

The nanoparticles were evaluated for antibacterial activity using the well diffusion method [30]. Pure cultures of *B. velezensis* were cultured in nutrient broth (NB) medium at 28 °C in a shaker incubator. After 48 h, 100 µL of the fresh bacterial culture was spread on nutrient agar (NA) plates, and the plates were left to stand for 10 min to allow the cultured cells to be absorbed. The antibacterial activity of the nanoparticles was assayed by punching 6 mm wells into the NA plates and adding 80 µL of SiO_2_ nanoparticle or CNT suspensions to individual wells with a micropipette. Distilled water was added to negative control wells. The plates were incubated at 28 °C for 24 h and then the diameters of the inhibition zones around the wells.

#### 2.5.8. Preparation of the ALG-Gelatin Microcapsules

An emulsion method was used for the microencapsulation of *B. velezensis*. All solutions and glassware used in these protocols were sterilized in an autoclave for 15 min at 121 °C. The bacteria were encapsulated according to the method described by Moradi-Pour et al. [5]. In this experiment, 10^10^ CFU/mL of *B. velezensis* cells was added to 100 mL of 2% ALG and 1.5% gelatin. The solution was then enriched with CNTs and SiO_2_ nanoparticles. The encapsulation was initiated by adding CaCO_3_ nanoparticles (2%) and soybean oil containing Span 80. The solution was homogenized and stirred on a magnetic stirrer for 15 min. Once the suspension became uniform, acetic acid (500 μL) was added, followed by stirring for a further 15 min. The encapsulation process was completed by adding CaCl_2_ (2%), and the capsule beads were collected by centrifugation (3000 rpm), washed with sterile physiological saline (0.9%), and stored at 4 °C.

#### 2.5.9. Efficiency of ALG-Gelatin Beads

The viability of the encapsulated bacteria was assessed by releasing the bacterial cells from the beads according to the method of Young et al. [31]. One gram of beads was added to 9 mL of phosphate buffer (0.1 M, pH 7.0) and left for 1 h. The colony-forming units (CFU/g) were then determined by culturing the released bacteria on NA plates at 28 °C for 24 h.

Encapsulation efficiency = (Number of viable cells encapsulated in capsules)/(Number of viable cells initially used to form capsules) × 100.

#### 2.5.10. Characterization of the ALG-Gelatin Microcapsules

The morphologies analysis of ALG-gelatin beads was characterized by scanning electron microscopy (TESCAN S8000, Brno, Czech Republic).

#### 2.5.11. X-ray Diffraction (XRD) Analysis

An X-ray diffractometer (D8-Advance, Bruker, Billerica, MA, USA) equipped with CuKα source (λ = 0.15406 nm) and operated at 40 kV/30 mA was used for XRD analysis. A 2θ angle range of 10–80 with 0.02°/s scan rate was applied for recording the samples.

#### 2.5.12. Viability of Microencapsulated Bacteria after One Year

The remaining viable *B. velezensis* cells entrapped in ALG-gelatin beads after one year were evaluated by storing the ALG-gelatin microencapsulated bacteria in a tube at room temperature. After one year, one gram of the formulation was immersed in 10 mL of sterile distilled water, shaken for one hour, and cultured on NA medium. After 24 h, the bacterial population was counted and reported.

#### 2.5.13. Assessment of *B. velezensis* Release and Viability in Soil

The bacterial release and viability in soil were assessed as described by Wu et al. [32]. The bacteria were first made resistant to rifampicin antibiotics [33]. After encapsulation, one g of dried microcapsules containing *B. velezensis* was buried in sterile soil and kept at room temperature for 60 days. During this period, soil moisture was maintained by spraying sterile distilled water on the soil surface. At various times, the number of bacterial cells released into the soil was determined by serial dilution of soil, spreading 100 µL of each dilution on NA, and incubating at 28 °C for 48 h. The bacterial colonies were counted and reported as CFU/g of dried ALG-gelatin microcapsules.

### 2.6. Greenhouse Experiments

#### 2.6.1. Preparation of the Bacterial Inoculant

A *B. velezensis* bacterial suspension for use in greenhouse experiments was prepared by suspending a fresh culture of bacteria in sterile distilled water and adjusting to 10^10^ CFU/mL by measuring optical density at 540 nm (Specord 250, Analytik Jena AG, Jena, Germany).

#### 2.6.2. Planting and Growth Conditions of Pistachio Plants

Sarakhs pistachio seeds were obtained from the Pistachio Research Institute of Rafsanjan (Rafsanjan, Iran) and planted in sterile soil according to the method of Moradi [34]. Four germinated seeds were planted in each pot containing two kg of sterile soil. When the plants reached two months of age, the pots were placed in a greenhouse at 25 to 27 °C, and irrigation was performed daily to keep the soil moisture constant for harvest. The seedlings were inoculated with *P. drechsleri*, and the *B. velezensis* bacterial strain was added to each kg of potting soil as a 10 mL suspension of a 24 h culture containing 10^10^ CFU/mL in sterile distilled water. Pots containing the nanoformulation treatments were inoculated by placing 10 g of *B. velezensis* microcapsules per kg of soil around the crown of the plant and covering it with soil. All pots were kept in a greenhouse at 25–30 °C temperature for 60 days.

#### 2.6.3. Growth Factor Measurement

The shoot lengths of each plant were measured using a ruler. The plant wet and dry weights were measured by removing the plants from the soil, washing and drying the aerial parts and roots, placing each separately in paper bags, drying in an oven at 50 °C for 48 h, and then weighing.

#### 2.6.4. Disease Control

After 60 days, the percentage of gummosis infection on pistachio plants was determined using the scale described by Thomas et al. [35].

### 2.7. Statistical Analysis

The data on bacterial release, viability, and growth factors were analyzed by one-way ANOVA. Significant SAS 9.1 (SAS Institute, Inc., Cary, NC, USA), Origin v 8.0 software (OriginLab Corporation, Northampton, MA, USA), and MestreNova software (Mestrelab Research, Santiago de Compostela, Spain) were used for data analysis, drawing XRD patterns, and NMR analysis, respectively.

## 3. Results and Discussion

### 3.1. Effect of Bacterial Strains on In Vitro Mycelial Growth of P. drechsleri

**Figure 2:**
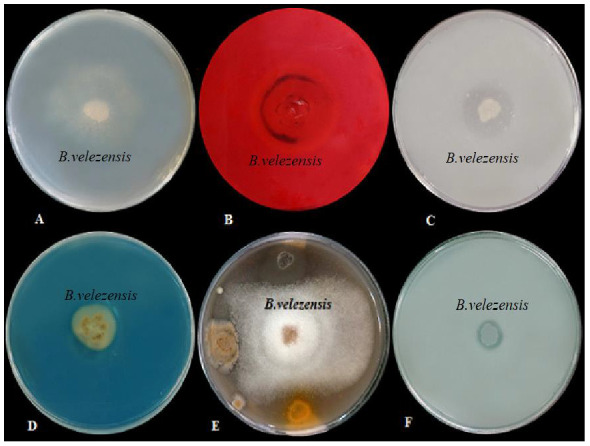
(**A**): Production of lipase; (**B**): Production of cellulose; (**C**): Production of protease enzyme; (**D**): Siderophore production; (**E**); Inhibition zone of *Phytophthora drechsleri*; (**F**): Phosphate solubilization activity.

After five days, the growth inhibition halo caused by the *B. velezensis* bacterial strain was 1.6 cm in diameter (Figure 2). The greatest inhibition effect on the radial growth of the fungal mycelium is due to the production of antibiotics by this bacterial species [36]. Important antibiotics produced by this bacterium can include iturin A, B, and E, bacillomycin, fengycin, and bacilysocin. Of course, no bacterium can produce all these antibiotics [37].

### 3.2. Cellulase Activities of B. velezensis

Incubation of individual plates with Congo red (1%) and NaCl solution (2 M) at 28 °C revealed cellulase production in the form of a yellowish halo (23 mm in diameter) around the *B. velezensis* colony (Figure 2). The beneficial effects of PGPR species such as *B. velezensis* on plants are due to the capability of the bacteria to inhibit soil-borne pathogens [38]. Cellulase production and the use of viable substrates in the rhizosphere are significant for controlling the rhizosphere competence [39]. PGPR inhibit the growth of plant pathogens by producing cell wall lytic enzymes, such as cellulase and chitinase, while also providing a suitable environment for the development of root systems [40].

### 3.3. Protease and Lipase Activities of B. velezensis

An in vitro screen was used to determine protease activity in *B. velezensis* inoculated on skim milk agar medium for 2 days. The bacterial strain showed positive protease production, indicated by a clear zone of 2 cm diameter around the colony (Figure 2). Lipase production activity was evaluated on tryptic soy broth medium containing 1% Tween80. A white precipitate around the bacterial colony indicated positive lipase activity (Figure 2). Lipases are widely produced by microorganisms and are of high industrial importance as they hydrolyze triglycerides to glycerol and free fatty acids and are useful in the pharmaceutical, food, textile, biodiesel, and detergent industries. Several bacteria produce lipases that can hydrolyze triglycerides [41]. Bacteria belonging to various genera are widely reported for the production of lipases [42]. Among them, the extracellular lipases produced by *Bacillus* spp. and *Pseudomonas* spp. are used in biotechnological applications [43]. Different *Bacillus* species that are able to produce proteases might be effective biocontrol agents [44,45].

### 3.4. Siderophore Production by B. velezensis

Screening of siderophore production by PGPR bacteria is typically conducted on chrome azurol S (CAS) agar medium. Spotting *B. velezensis* bacteria onto CAS agar plates resulted in the production of orange halos (17 mm diameter) around the colony, indicating siderophore production (Figure 2). In the CAS agar medium test, a complex of CAS/hexadecyl trimethyl ammonium bromide and Fe^3+^ serves as an index. When a potent iron chelator, such as a siderophore (produced by bacteria), removes the iron from the dye, the dye color changes from blue to orange [46]. Bacterial siderophores have a positive relationship with plant growth promotion; therefore, one of the main reasons for screening PGPRs for agricultural management is their siderophore production. Many reports describe the relationship between siderophore-producing PGPRs and plant growth-promoting effects [47,48,49].

### 3.5. Solubilization of Phosphorus

Phosphorus is an essential mineral for plant growth, and the lack of it generally limits the growth of plants. Although agricultural soils contain much phosphorus, the amount available for use by plants is a low ratio of the total [50]. The observation of a 13 mm diameter halo formed around the bacterium colony confirmed that the *B. velezensis* strain had P-solubilization potential (Figure 2). The P-solubilization ability of bacterial strains can stimulate lateral root formation and improve nutrient uptake, thereby increasing plant resistance to pathogens [51].

### 3.6. Determination of IAA Production

*B. velezensis* produced 2.41 g/mL of IAA (indolyl-3-acetic acid) in the medium. The production of IAA is necessary for the promotion of plant growth by PGPR. Tsavkelova et al. [52] showed that microbial IAA producers had an essential role in stimulating orchid germination. The beneficial effects of PGPR on plants are mainly related to the production of phytohormones, such as auxins such as IAA [53]. In the present study, the observed plant growth simulation could be related to IAA secretion and phosphate solubilization.

### 3.7. ^1^H NMR Spectra of the Extracted ALG

**Figure 3:**
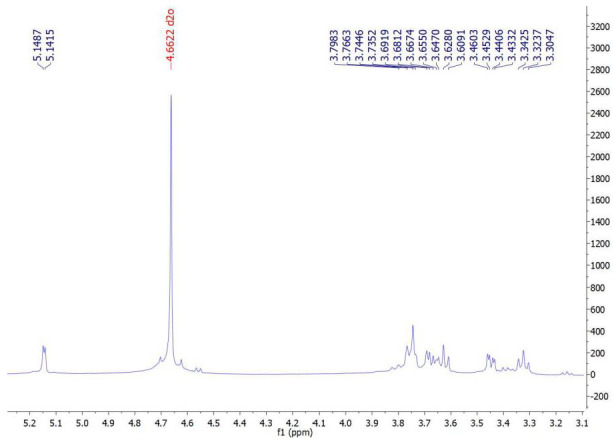
^1^H NMR spectrum of extracted ALG in D_2_O and its characteristic signals.

**Figure 4:**
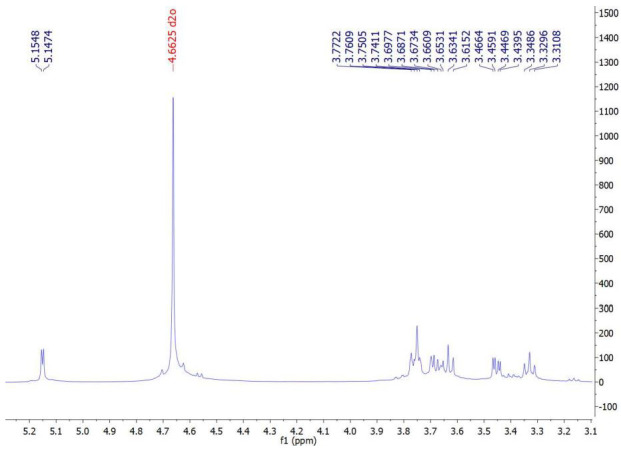
^1^H NMR spectrum of a commercial ALG in D_2_O and its characteristic signals.

Examination of the ^1^H NMR spectrum of the extracted ALG sample with the commercial sample showed an exact correspondence of their proton signals. Therefore, the extraction and purification of ALG from algae was conducted correctly. The ^1^H NMR spectrum of the extracted ALG showed signals related to the anomeric protons of L-guluronosyl and D-mannuronosyl residues, well separated at 4.66 and 5.14 ppm, respectively. These signals were the same as in the commercial sample (also 4.66 and 5.14 ppm), again confirming the accuracy of the ALG extraction (Figure 3 and Figure 4).

### 3.8. FTIR Spectra of ALG Samples

**Figure 5:**
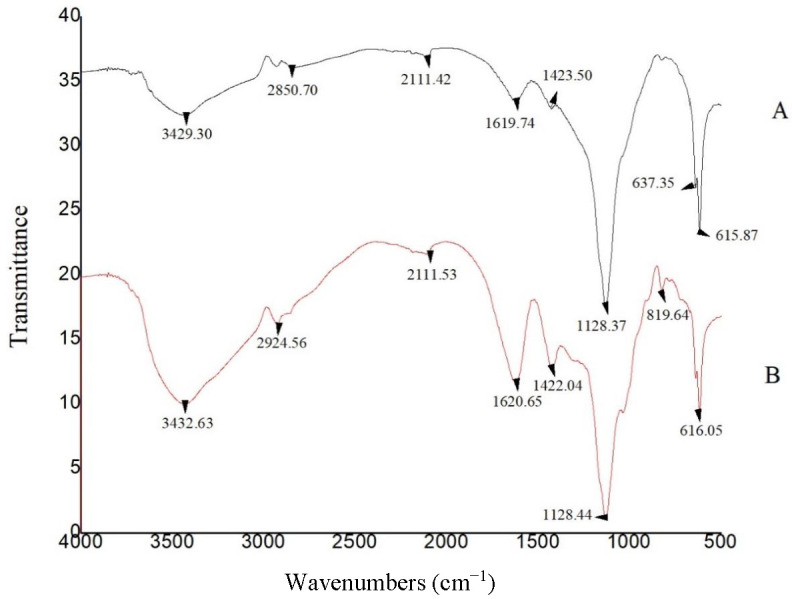
FTIR spectra of A: extracted ALG; B: commercial ALG (Sigma).

The FTIR spectra of the extracted and commercial ALG samples are shown in Figure 5. The absorption band related to aliphatic C–H stretching vibrations appeared near 2850 cm^−1^ in the extracted ALG and around 2924 cm^−1^ in the commercial sample. Bands of COO^−^ asymmetric stretching vibration were observed at 1619 cm^−1^ and 1620 cm^−1^ for the extracted and commercial samples, respectively. The sharp absorption band related to the symmetric COO^−^ stretching vibration appeared at 1423 cm^−1^ and 1422 cm^−1^ for the extracted and commercial samples, respectively. The bending C–H vibration appeared at 1152 cm^−1^ in both samples. Therefore, based on the ^1^H NMR and FTIR results, the extracted ALG was structurally consistent with commercial ALG.

### 3.9. The Physiochemical Properties of ALG

The physicochemical properties of ALG extracted from *Macrocystis pyrifera* and the commercial ALG (Sigma) are presented in Table 1. The weight average molecular weights (Mw) of commercial ALG and ALG extracted from algae were 1.78 × 10^5^ and 1.39 × 10^5^, respectively. Therefore, the physicochemical properties were similar in both samples.

### 3.10. Efficiency of Nanoparticle Encapsulation of B. velezensis

The absence of a halo of growth inhibition around *B. velezensis* bacterial colonies exposed to nanoparticles (SiO_2_ and CNT) indicated no adverse effects of the nanoparticles on bacterial growth and viability. Numerous studies reported a positive effect of some nanoparticles on plant growth [5,6,9,54,55,56,57,58,59]. Therefore, the use of nanoparticles in the encapsulation of bacteria is expected to further improve plant growth. Some nanoparticles have intense antibacterial activities; therefore, they must be tested to ensure that they are not antibacterial.

### 3.11. X-ray Diffraction Analysis

XRD analysis was used to investigate the crystallinity of the ALG, gelatin, and ALG/gelatin microcapsules. The gelatin analysis revealed crystalline reflection at 2θ = 20.8°, which agreed with the stable gelatin crystals reported by Phadke et al. [60]. The analysis of the ALG spectra indicated two reflections at 2θ = 21.5° and 2θ = 13.4°. Thu and Ng [61] obtained similar results under the same conditions. However, when ALG was mixed with gelatin in the present formulation, the reflections were shifted to 2θ = 31.6° and 2θ = 43.5°. This was probably due to the presence of CaCl_2_ crystals that formed during the encapsulation process. The disappearance of the typical reflections of ALG and gelatin showed that the electrostatic interaction between the carboxyl group of ALG and the amino group of gelatin weakens the structure.

**Figure 6:**
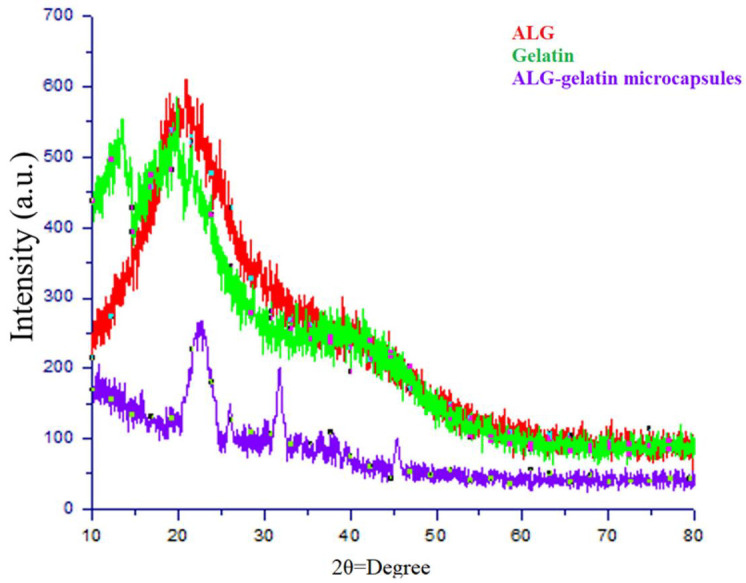
XRD patterns of ALG, gelatin, and ALG-gelatin microcapsules.

Nallathambi et al. [62] also reported that crystal peaks related to the amorphous nature of SiO_2_ nanoparticles should appear at 2θ = 22°, and this was observed in the diffractogram. Chen and Oh [63] reported a reflection for pure multi-walled CNTs at 2θ = 25.9°. The XRD pattern of the beads formulated in the present study showed a similar reflection, presumably related to the presence of CNTs in the formulation (Figure 6).

### 3.12. Microscopy Study of ALG-Gelatin Microcapsules

**Figure 7:**
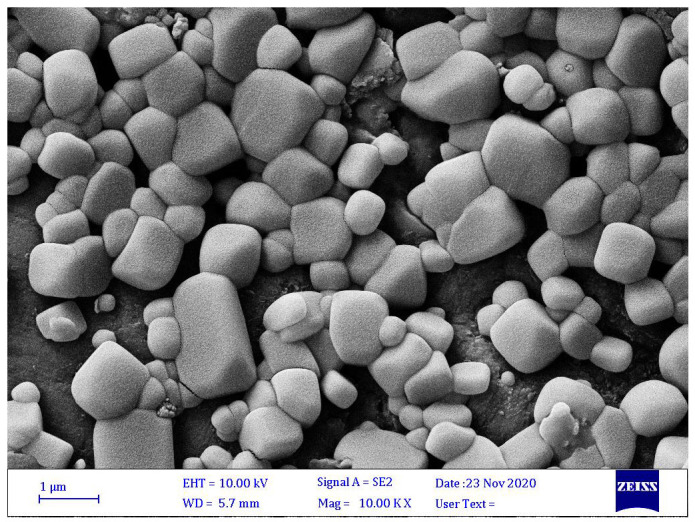
SEM of microcapsules with *B. velezensis*. Adapted from [64].

**Figure 8:**
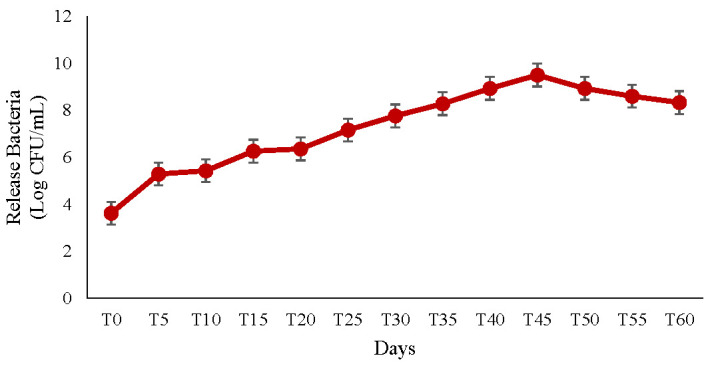
Release of *B. velezensis* from microcapsules prepared with ALG and gelatin.

The average particle size of the microcapsules probably depended on the efficiency and stability of microencapsulation [40]. Microcapsules with larger particle sizes commonly offer more protection for the core material than is afforded by smaller ones; however, larger particles show little dispersion in the final products. Hence, powders with too fine a grain can tend to have empty particles with no core materials entrapped within them, so their efficiency is often low. Optimal particle size is therefore important both for protection and for maximal encapsulation of *B. velezensis* and to ensure an adequate distribution of microcapsules in the products. The SEM image (Figure 7) shows that the ALG-gelatin microcapsules were irregularly shaped with cubic motifs and had a large size variation. This irregular shape can affect the entrapment and release of the bacterial strain (Figure 8).

### 3.13. Assessment of the Extent of Bacterial Release and Viability in Soil

The ANOVA results indicated a significant effect of release time on the release of the bacteria from the microcapsules and their survival rate (*p* ˂ 0.01). The number of bacteria released from the ALG-gelatin microcapsules, as determined by the plate count method on NA plates [32], increased quickly until the 50th day after exposure of the formulation to moisture. The highest release percentage was observed on the 50th day, and the population was reduced dramatically afterward. This decrease probably indicated a depopulation of released bacteria because a number of them began to die in the capsules (Figure 8).

### 3.14. Evaluation of the B. velezensis Survival Rate in Microcapsules after One Year of Storage

One year after storage at room temperature, the encapsulated *B. velezensis* population, with an initial population of 10^10^ CFU/mL, was reduced to a population of approximately 10^7^ CFU/mL remaining in the capsules. Therefore, the storage of this formulation under normal room conditions and in the absence of moisture is sufficient to maintain a large bacterial population within the capsules.

### 3.15. Greenhouse Experiments

The use of microcapsules prepared with *B. velezensis* for pathogen control in greenhouse conditions resulted in a significant increase in pistachio plant growth and substantial control of pistachio gummosis. The *B. velezensis* microcapsules showed the highest ability (93.66%) to reduce *P. drechsleri* infection, compared with disease control levels of 75% and 25% in control plants treated with uncoated bacteria or with microcapsules without bacteria, respectively. The growth of plants treated with encapsulated bacteria was significantly enhanced (*p* ˂ 0.01) over the control treatments. Shoot lengths and shoot and root fresh and dry weights were greater in plants treated with microcapsules than in the control plants.

Pistachio plants inoculated with bacteria in microcapsules initially grew more slowly than those inoculated with free bacteria. This might reflect a gradual release of the bacteria from the microbeads. The inoculation of pistachio plants with microencapsulated bacteria or with free bacterial strains resulted in increased growth compared with uninoculated control plants. The shoot lengths of plants treated with *B. velezensis* microcapsules showed a significant (*p* ˂ 0.05) increase compared to the lengths of pistachio plants treated with the free bacteria (Table 2). The plants treated with bacteria showed increased shoot and root weights compared to the untreated control plants.

In this research, we investigated the plant growth-promoting (PGP) activity of *B. velezensis* in pistachio plants, and we examined the benefits of encapsulating the PGPR in microcapsules prepared with a biopolymer (ALG-gelatin) and nanoparticles (SiO_2_ and CNT). A high bacterial survival rate was maintained in the microcapsules (10^7^ CFU/mL), with a minimum bacterial loss, during storage for one year. Holding the *B. velezensis* microcapsules at room conditions and protected from humidity could be sufficient for long-term storage. The formulation is designed in a way that when a signal, such as moisture, stimulates the polymers, the bacteria begin to exit gradually from the capsule wall. The release stops immediately upon moisture reduction, and the bacteria remain within the capsule until the signal is activated again.

The introduction of nanotechnology into agriculture is anticipated, and nanoparticles play an important role in increasing plant growth [65]. Since living agents are the active substances in biological products, each formulation must have sufficient durability. Therefore, control is needed at all steps of formulation and long-term storage to maintain biological activity [65]. The results of the present study showed that bacteria, and the microcapsules they are enclosed in, can both increase plant growth. In general, the pistachio plants inoculated with all treatments under greenhouse conditions showed increased growth.

Among the studied treatments, the bacteria in microcapsules had the most significant positive effect on plant growth parameters, whereas treatments with bacteria without coatings and with microcapsules alone without bacteria showed a lesser effect on growth parameters. Therefore, in addition to the gradual release and high survival rate of encapsulated bacteria, which makes their biological effects more efficient, another possibility is that the nanoparticles incorporated into the capsule wall may act synergistically with bacterial metabolites. Due to their small size, CNTs easily cross biological barriers, bind to beneficial bacterially produced metabolites, and facilitate their entry into the plant, thereby making the compounds more easily available to the plant [66,67]. Among the compounds that play important roles in increasing plant growth parameters, the hormone auxin is produced in large quantities by the bacteria studied in this research, and it penetrates the plant via functionalized CNTs to promote longitudinal growth.

Drought stress is an important factor limiting crop production in arid and semi-arid areas where pistachios are grown [68], and some researchers believe that Si is an essential element for plants. Liang et al. [69] and Pei et al. [70] showed that Si supplementation could reduce the adverse effects of oxidative stress and cause resistance to biotic and abiotic plant stressors [68]. Si absorption by plants occurs more readily when supplied in the form of nanoparticles than as free Si [71]. Nanosilica particles also increase plant resistance to diseases and stimulate their physiological activities. These nanoparticles improve the water uptake efficiency of the plant by affecting the wetness of the vascular tissue and increasing the water transfer rate. Haghighi et al. [72] treated salt-stressed tomato seeds with nanosilica and showed a decrease in the harmful effects of salinity on germination, root length, and dry weight of the seedlings. Microencapsulated *B. velezensis* showed longer survival than free bacteria and had a higher efficacy for enhancing plant growth under greenhouse conditions. The bacterial viability provided by this encapsulation technique is mainly due to the dual benefit of nanoparticles in terms of their chemical properties and plant growth enhancement.

## 4. Conclusions

The results presented here show that ALG-gelatin nanocomposites are effectively able to encapsulate *Bacillus velezensis* for the control of pistachio gummosis. The results of XRD analyses revealed that ALG and gelatin undergo electrostatic interactions. Biological evaluations showed that the microcapsules have growth-promoting effects on pistachios. Release experiments confirmed the sustained release of the encapsulated *Bacillus velezensis* from the microcapsules. The encapsulated formulation showed a higher biological effect than unencapsulated bacteria. Overall, the nanoformulations used to protect bacteria against environmental conditions can also be used to control plant pathogens. The present chemical methods used to control pistachio gummosis cause environmental problems. Using this formulation may therefore represent a new step in the biological control of this disease.

## Figures and Tables

**Table 1:** Physicochemical properties of ALG extracted from *Macrocystis pyrifera* and commercial ALG (Sigma).

Sample	Color	Density (g/cm^3^)	Mw	M/G Ratio *
ALG extracted from *Macrocystis pyrifera*	Light brown	1.6	1.78 × 10^5^	1.42
ALG Sigma (A2033)	Light brown	1.62	1.39 × 10^5^	1.57

* M/G is the mannuronic acid/guluronic acid molar ratio.

**Table 2:** Effects of *B. velezensis* inoculation as a free cell preparation and as a microencapsulated preparation on growth parameters in pistachio plants.

Treatment	Root Fresh Weights	Root Dry Weights	Shoot Fresh Weights	Shoot Dry Weights	Plant Height
*B. velezensis* microcapsules	2.35 ^a^	0.79 ^a^	3.26 ^a^	1.29 ^a^	16.00 ^a^
*B. velezensis* microcapsules + *P. drechsleri*	2.21 ^b^	0.69 ^a^	3.13 ^a^	1.14 ^a^	14.33 ^b^
Free *B. velezensis*	2.19 ^b^	0.68 ^a^	3.09 ^a^	1.12 ^a^	13.33 ^b^
Free *B. velezensis* + *P. drechsleri*	1.81 ^c^	0.38 ^b^	2.65 ^b^	0.64 ^b^	11.66 ^c^
Microcapsules without bacteria	1.75 ^c^	0.3b ^c^	2.51 ^b^	0.62 ^b^	10.66 ^c^
*P. drechsleri*	1.29 ^d^	0.21 ^c^	2.00 ^c^	0.32 ^c^	6.66 ^e^
Control	1.75 ^f^	0.25 ^c^	2.48 ^b^	0.53 ^bc^	8.33 ^d^

Different letters indicate significant differences at *p* ≤ 0.05.

## Data Availability

Not applicable.
